# Sequential transcriptome profiling: comparative analysis of normal and canine lymphoma preceding detailed T-cell and B-cell subtype comparison

**DOI:** 10.3389/fvets.2024.1473421

**Published:** 2025-01-22

**Authors:** Yeji Kim, Jihyun Kim, Yunji Song, Keunhwan Jang, Se Eun Kim, Ha-Jung Kim

**Affiliations:** ^1^Department of Veterinary Internal Medicine, College of Veterinary Medicine, Chonnam National University, Gwangju, Republic of Korea; ^2^BK21 FOUR Program, Chonnam National University, Gwangju, Republic of Korea; ^3^Department of Veterinary Surgery, College of Veterinary Medicine, Chonnam National University, Gwangju, Republic of Korea; ^4^Biomaterial R&BD Center, Chonnam National University, Gwangju, Republic of Korea

**Keywords:** lymphoma, T-cell, B-cell, transcriptome, microarray, heterogeneity, dog

## Abstract

**Introduction:**

As the lifespan of companion animals extends, the incidence of tumor also increases. Among these tumors, lymphoma is reported as the most prevalent hematopoietic tumor with a 80-90% prevalence rate. Ongoing research spans multiple domains, aiming to uncover novel therapeutic targets, including small molecular weight inhibitors, antibody treatments, and subtype-specific selective agents.

**Methods:**

Transcriptional profiling was performed on canine lymphoma samples to identify genes and functional pathways associated with pathogenesis, treatment response, and prognosis. Additionally, genes with potential relevance to the clinical characteristics of T-cell lymphoma (TCL), which is characterized by a low treatment response and poor prognosis, were identified through a comparative analysis of different lymphoma subtypes.

**Results:**

Within the canine lymphoma group, HERC5 showed consistent upregulation, a gene similarly implicated in human acute myeloid leukemia but previously no reports exist. Additionally, noteworthy genes, including IKZF2, CCL4, SAA1, and CD40, exhibited differential expression in the TCL group compared to the B-cell lymphoma (BCL) group.

**Discussion:**

The upregulation of HERC5 may impact on canine lymphoma pathogenicity. Furthermore, the upregulation of IKZF2, CCL4, and SAA1, along with the downregulation of CD40, may contribute to adverse clinical characteristics of TCL in dogs.

## Introduction

Lymphoma is a prevalent hematopoietic tumor that results from the malignant transformation of lymphocytes (1). It exhibits diverse subtypes, classified based on morphology, immunophenotype, and molecular variations (2, 3). To differentiate between T-cell and B-cell types, complementary diagnostic tests, such as flow cytometry and immunohistochemistry, are conducted (2, 3). Notably, B-cell lymphoma (BCL) generally demonstrates a more favorable response to treatment and prognosis compared to T-cell lymphoma (TCL) in dogs (4), with previous reports indicating lower complete remission rates and shorter survival times, approximately 40%, in dogs with TCL compared to in those with BCL (5, 6). Because the prognosis varies depending on the type of lymphoma, it is important to differentiate between BCL and TCL (5, 6).

Recently, numerous studies have been conducted in the field of genetics to characterize subtypes, diagnose, and understand the prognosis of lymphoma (7–9). “Transcriptome” encompasses all transcripts, including mRNAs, non-coding RNAs, and small RNAs, present within a cell at a given developmental stage or physiological state (7). Analyzing splicing patterns, post-transcriptional modifications, and expression levels of the transcriptome in a particular environment is crucial for understanding genome functionality and gaining insights into tumor biology (7–9). A protocol has been established to classify melanoma, osteosarcoma, lung cancer, B-cell lymphoma, and T-cell lymphoma using canine transcriptome data (8). The analysis identified a total of 625 genes exhibiting significant differences, and Kyoto Encyclopedia of Genes and Genomes (KEGG) analysis indicated 11 pathways associated with the progression of lymphoma in dogs (9). Further research is necessary to accurately diagnose canine lymphoma and predict prognosis. While numerous studies have focused on the transcriptome of B-cell lymphoma (BCL) compared to normal samples in dogs, there is a paucity of research examining the differences between T-cell lymphoma (TCL) and B-cell lymphoma (BCL) (9–11).

The current study aims to compare transcriptomic profiles between normal samples and lymphoma, including each lymphoma subtype, to analyze gene expression differences. Furthermore, it seeks to explore associated biochemical pathways for lymphoma diagnosis and prognosis.

## Materials and methods

### Study population and design

The study included a population of 13 client-owned dogs diagnosed with multicentric lymphoma and 7 dogs without lymphoma. The clinical characteristics of each participant are detailed in Table 1. The 13 dogs in the lymphoma group were referred for internal medicine evaluation to Chonnam National University Teaching Hospital in 2020–2022 (Identification code No. CNU IACUC-YB-2021-166, No. CNU IACUC-YB-2022-121).

**Table 1:** Clinical characteristics of dogs included in this study.

No.	Breed	Age (Years)	Sex	Diagnosis	TNM stage	Immuno-phenotyping
Lymphoma group						
L1	Maltese	17	M	Multicentric lymphoma	4b	B
L2	Mixed	14	F	Multicentric lymphoma	4b	T
L3	Poodle	9	CM	Multicentric lymphoma	4b	B
L4	Mixed	6	M	Multicentric lymphoma	4b	T
L5	Chihuahua	7	CM	Multicentric lymphoma	4b	T
L6	Maltese	8	CM	Multicentric lymphoma	3b	T
L7	Maltese	11	CM	Multicentric lymphoma	5a	T
L8	Maltese	8	F	Multicentric lymphoma	5a	B
L9	Bichon Frise	5	SF	Multicentric lymphoma	4b	B
L10	Poodle	4	CM	Multicentric lymphoma	4a	T
Control group						
C1	Beagle	1.5	M	Healthy	–	–
C2	Beagle	1.5	F	Healthy	–	-
C3	Beagle	1.5	F	Healthy	–	–
C4	Beagle	1.5	F	Healthy	–	–
C5	Beagle	1.5	F	Healthy	–	–
C6	Beagle	1.5	F	Healthy	–	–
C7	Beagle	1.5	F	Healthy	–	–

CM, castrated male; SF, spayed female.

The staging of lymphoma cases was performed in accordance with the World Health Organization TNM classification system for canine lymphoma (12). Among the dogs diagnosed with lymphoma, 10% (1/10) were classified as stage III, 70% (7/10) as stage IV, and 20% (2/10) as stage V. Additionally, 60% (6/10) of dogs were classified as TCL, while 40% (4/10) were identified as BCL (Table 2).

**Table 2:** Classification of healthy, lymphoma and the World Health Organization’s clinical staging system.

Group		N	%
Healthy control		7	41.2
Lymphoma		10	58.8
Lymphoma immunophenotype	T cell	6	35.3
	B cell	4	23.5
Lymphoma stage	I	0	0
	II	0	0
	III	1	5.8
	IV	7	41.2
	V	2	11.8
Lymphoma substage	a	3	17.6
	b	7	41.2

### Sample collection

For dogs with lymphoma, lymph node tissues were collected through either ultrasound-guided fine needle aspiration or biopsy procedures on enlarged peripheral or abdominal lymph nodes at initial diagnosis. The collected tissues were preserved in RNAprotect Tissue Reagent (Qiagen, Hilden, Germany) and stored at −80°C until further analysis.

In the control group, one dog underwent anesthesia, and tissue samples were collected from six dogs post-euthanasia. Following a thorough disinfection procedure, an incision was made in the left hind leg muscle to access and isolate the left popliteal lymph node. The lymph node and adjacent fat tissue were separated, and then preserved in a similar manner to the lymphoma samples.

### Anesthesia and euthanasia

All dogs undergoing biopsy for the diagnosis of lymphoma, as well as one dog in the control group, were anesthetized. For pre-medication, which included sedation and induction, glycopyrrolate (5 μg/kg, SC; Mobinul^®^ Injection, Myungmoon, South Korea), medetomidine (5–10 μg/kg, IM; Tomidin^®^ Injection, Provet Veterinary Products, Turkey), and alfaxalone (1.5–2 mg/kg, IV; Alfaxan^®^ Injection, JUROX, Australia) were administered. During the surgical procedure, respiratory anesthesia was maintained with isoflurane (2–3%, inhalation; Terrel™ Solution, Piramal Critical Care, USA).

Furthermore, for the sedation and induction of six dogs in the control group, medetomidine (5–10 μg/kg, IM; Tomidin^®^ Injection, Provet Veterinary Products, Turkey) and alfaxalone (1.5–2 mg/kg, IV; Alfaxan^®^ Injection, JUROX, Australia) were utilized. Subsequently, 20% potassium chloride (20 mL/body, IV; Potassium Chloride-40® Injection, DaeHan Pharm, South Korea) was employed as part of the euthanasia protocol.

### Transcriptome analysis

A total of 13 dogs diagnosed with lymphoma and 7 dogs without lymphoma were included in the transcriptome analysis. Three of the lymphoma samples did not provide sufficient RNA for analysis and therefore excluded from the study. The isolation of total RNA from each tissue was performed using the Transzol-based RNA extraction protocol.

The concentration and purity of total RNA were assessed utilizing the ND-2000 Spectrophotometer (NanoDrop, Wilmington, USA), while the integrity was evaluated with the Agilent RNA 6000 Nano Kit on a 2,100 Bioanalyzer (Agilent Technologies, Palo Alto, USA). For RNA labeling and hybridization, the Agilent One-Color Microarray-Based Gene Expression Analysis protocol (Agilent Technology, Version 6.5, 2010) was utilized. The labeled complementary RNAs (cRNAs) were purified using the RNAeasy Mini Kit (Qiagen). The hybridization solution was dispensed onto the gasket slide and then assembled onto the Agilent Unrestricted AMADID Release GE 4x44K (Canine V2). The hybridized array was then scanned using the Agilent Microarray Scanner D (Agilent Technologies). Data extraction from the microarray was conducted using the Agilent Feature Extraction software version 11.0 (Agilent Technologies).

Subsequently, the raw data for each gene were automatically summarized according to the Agilent feature extraction protocol, resulting in the generation of a raw data text file that contained expression data for each gene probed on the array. A comparative analysis of expression profiles among analogous samples was conducted utilizing the Agilent Canine GE 4X44k v2 data.

### Differential expression and functional analysis

Differential expression analysis between the control and lymphoma groups was conducted using R version 3.3.2. *p*-values underwent Benjamini and Hochberg methods to account for multiple testing. Significance was defined as an FDR-adjusted p-value (P) <0.05 and a log2 fold change ≥2 for the selection of differentially expressed genes (DEGs). Subsequently, DEGs were analyzed for gene enrichment and functional annotation utilizing the KEGG. Data analysis and visualization of DEGs were also performed using R version 3.3.2.

### Statistical analysis

To evaluate the normality of the variables, the Shapiro–Wilk test was used, and the data were expressed as mean ± standard error of the mean. The statistical significance of the expression data was assessed through fold change analysis and independent t-tests, with the null hypothesis positing no difference between groups. The false discovery rate (FDR) control was achieved by adjusting *p*-values using the Benjamini–Hochberg methods. Hierarchical cluster analysis, based on complete linkage and Euclidean distances, was conducted for DEGs. Significance was set at *p* < 0.05.

## Results

### Identification of DEGs in the lymphoma group compared to the control group

**Figure 1:**
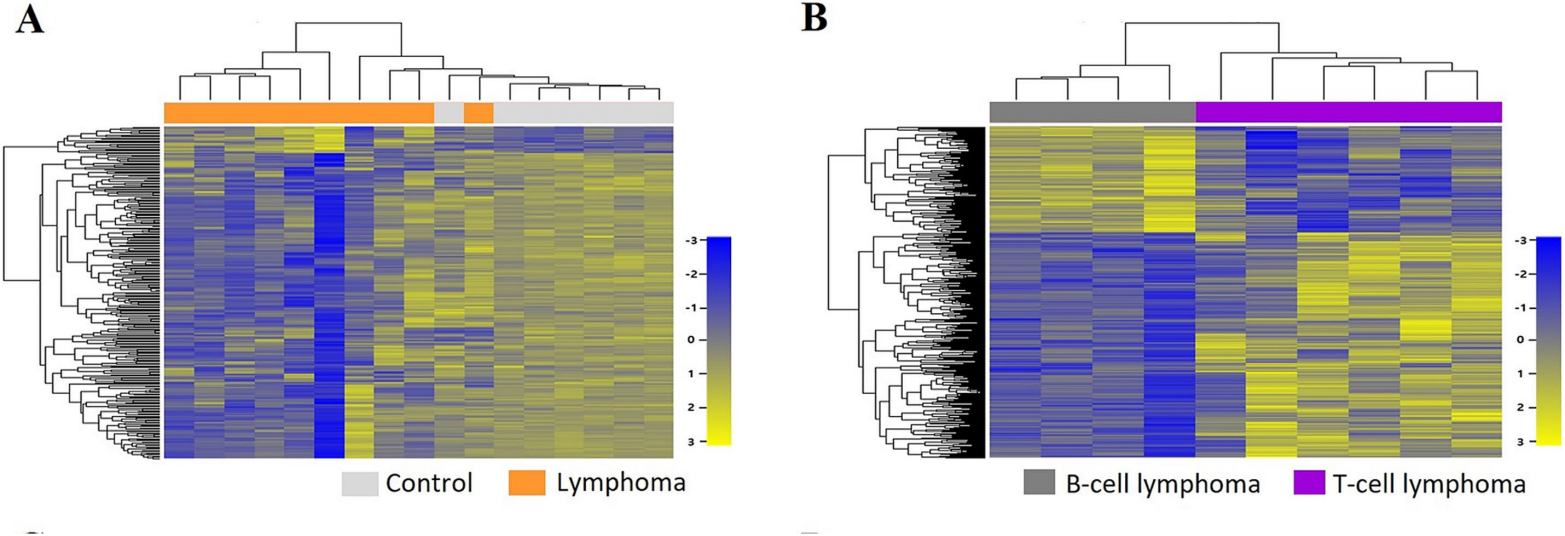
Two-way hierarchical clustering heatmap of differentially expressed genes (DEGs) identified in the lymphoma group compared with the control group **(A)** and in the T-cell lymphoma (TCL) group compared with the B-cell lymphoma (BCL) group **(B)**.

A total of 43,603 genes were identified through transcriptomic analysis. Based on the set criteria, a total of 282 DEGs were identified in lymphoma vs. control groups, which included 22 upregulated genes and 260 downregulated genes. To visually depict the distinct gene expression patterns, a hierarchical clustering heatmap was constructed, illustrating the divergence between the lymphoma and control groups (Figure 1A).

Among the DEGs, five most highly expressed DEGs were *MX1* (MX dynamin-like GTPase 1), *HERC5* (HECT and RLD domain-containing E3 ubiquitin protein ligase 5), *OAS1* (2′-5′-oligoadenylate synthetase 1), and *DDX58* (DEAD box polypeptide 58). In contrast, the five DEGs with the lowest expression levels were *CAMP* (Cathelicidin antimicrobial peptide), *NMB* (Neuromedin B), *JAM2* (junctional adhesion molecule 2), *IL7R* (interleukin 7 receptor), and *GRAP2* (GRB2-related adaptor protein 2). Detailed information is available in Table 3, and a visual representation in Supplementary Figure S1A.

**Table 3:** The top five upregulated and downregulated DEGs in the lymphoma group vs. the control group.

Upregulated DEGs			Downregulated DEGs		
Gene	Fold change	*p*-value	Gene	Fold change	*p*-value
*MX1*	3.321125	0.029416233*	*CAMP*	−5.947344	0.035509294*
*MX1*	2.963822	0.039729606*	*NMB*	−5.362066	0.002581536**
*HERC5*	2.457227	0.03979437*	*JAM2*	−5.232453	0.003124124**
*OAS1*	2.390920	0.010895196*	*IL7R*	−3.664609	0.015739092*
*DDX58*	2.109487	0.025624632*	*GRAP2*	−2.156769	0.016341599*

Values with statistical significance are indicated in bold. *p < 0.05, **p < 0.01, ***p < 0.001, ****p < 0.0001.

### Function and pathway enrichment analysis of the DEGs in the lymphoma group

In the lymphoma group, 21 significant KEGG pathways were identified. The ten most prominent pathways included hematopoietic cell lineage, measles, T-cell receptor signaling, cancer pathways, cytokine–receptor interaction, primary immunodeficiency, viral myocarditis, PI3K–Akt signaling, arrhythmogenic right ventricular cardiomyopathy, and coronavirus disease, as presented in Table 4.

**Table 4:** The top 10 KEGG pathways in the lymphoma group compared with the control group.

Map name	*p*-value
Hematopoietic cell lineage	2.48713E-06****
Measles	1.97843E-05****
T-cell receptor signaling pathway	0.000110752***
Pathways in cancer	0.000302371***
Cytokine–cytokine receptor interaction	0.000512224***
Primary immunodeficiency	0.003090996**
Viral myocarditis	0.006842236**
PI3K–Akt signaling pathway	0.011109756*
Arrhythmogenic right ventricular cardiomyopathy	0.015084018*
Coronavirus disease - COVID-19	0.016504936*

Values with statistical significance are indicated in bold. *p < 0.05, **p < 0.01, ***p < 0.001, ****p < 0.0001.

### Identification of DEGs in the TCL group compared with the BCL group

In the comparative analysis of the control group and the lymphoma groups, as well as between the control group and each lymphoma subtype, a greater number of DEGs exhibited downregulation rather than upregulation. Conversely, when comparing the comparison between the TCL group and the BCL group, there was higher incidence of upregulated genes (*n* = 378) compared to downregulated genes (*n* = 177), resulting in a total of 555 DEGs. The expression patterns of these DEGs in the TCL group compared to the BCL group are illustrated in a hierarchical clustering heatmap (Figure 1B).

Among the DEGs identified, the five most highly expressed DEGs were *IKZF2* (Ikaros family zinc finger 2, Helios), *LOC102155886* (serum amyloid A protein-like), *CCL4* (C-C motif chemokine ligand 4), *IL1R2* (interleukin 1 receptor, type II), and *SAA1* (serum amyloid A1). In contrast, the five *DEGs* with the lowest expression levels included *LOC484343* (sialic acid-binding immunoglobulin-like lectin 10), *PLEKHA5* (pleckstrin homology domain-containing family A member 5), *GRK4* (G protein-coupled receptor kinase 4), *MYRIP* (myosin VIIA and Rab-interacting protein), and *CD40* (cluster of differentiation 40 molecule). Further details are available in Table 5, and a visual representation is provided in Supplementary Figure S1B.

**Table 5:** The top five upregulated and downregulated DEGs in the TCL group vs. the BCL group.

Upregulated DEGs			Downregulated DEGs		
Gene	Fold change	*p*-value	Gene	Fold change	*p*-value
*IKZF2*	11.254691	0.043498788*	*LOC484343*	−6.026889	0.040092473*
*LOC102155886*	11.112800	0.000736212***	*PLEKHA5*	−5.760278	0.011403542*
*CCL4*	10.141287	0.025482128*	*GRK4*	−5.165037	0.041780491*
*IL1R2*	8.284900	0.04620561*	*MYRIP*	−3.834566	0.046735826*
*SAA1*	8.239238	0.00204142**	*CD40*	−2.824743	0.048353666*

Values with statistical significance are indicated in bold. *p < 0.05, **p < 0.01, ***p < 0.001.

### Function and pathway enrichment analysis of DEGs in the TCL group

The KEGG pathway analysis revealed a total of 85 significant pathways within the TCL group. The ten most prominent pathways identified include: human T-cell leukemia virus 1 infection, focal adhesion, lipid and atherosclerosis, pathways in cancer, axon guidance, transcriptional misregulation in cancer, fluid shear stress and atherosclerosis, cell adhesion molecules, Yersinia infection, and lysosome (Table 6).

**Table 6:** The top 10 KEGG pathways in the TCL group compared with the BCL group.

Map name	*p*-value
Human T-cell leukemia virus 1 infection	6.77471E-08****
Focal adhesion	3.25781E-07****
Lipid and atherosclerosis	4.08606E-07****
Pathways in cancer	6.51299E-07****
Axon guidance	1.42261E-06****
Transcriptional misregulation in cancer	1.6279E-06****
Fluid shear stress and atherosclerosis	1.92482E-06****
Cell adhesion molecules	2.13947E-06****
*Yersinia* infection	1.21574E-05****
Lysosome	1.41089E-05****

Values with statistical significance are indicated in bold. *p < 0.05, **p < 0.01, ***p < 0.001, ****p < 0.0001.

## Discussion

The current study conducted a comparative analysis between lymphoma and control subjects, and sequential transcriptome analysis of TCL and BCL in dogs. In the comparison between lymphoma dogs and controls, we found that the upregulation of HERC5 may be associated with the pathogenic mechanisms underlying canine lymphoma. Furthermore, we identified the upregulation of IKZF2, CCL4, and SAA1, as well as the downregulation of CD40 in dogs with TCL compared to those with BCL, all of which were reported to be associated with shorter survival times in humans.

Among the DEGs that exhibited significant expression alternations in the lymphoma group compared with the control group, it has been reported that elevated expression of MX1 is significantly associated with higher tumor grade and lymphovascular invasion (13), elevated expression of OAS1 correlates with higher tumor grade and reduced overall survival (14, 15), and elevated expression of DDX58 is associated with increased microsatellite instability and tumor mutation burden, which are related to decreased overall survival and progression-free survival (16), indicating a poor prognosis in human cancers. This study also confirmed high expression of these genes in the lymphoma group.

A recent study has indicated a downregulation of HERC5 expression in human acute myeloid leukemia; however, there are no related reports within the field of veterinary medicine (17). Conversely, IL7R has been indicated as a favorable prognostic maker and a potential target for immunotherapy in human lung adenocarcinoma (18). This protein encoded by *IL7R* plays a critical role in inhibiting tumor growth by modulating the proportion of immune infiltrating cells within the tumor’ s immune microenvironment. Notably, low expression of *IL7R* has been correlated with increased tumor growth and lower survival rates (18). In our study, we confirmed lower expression of *IL7R* in the lymphoma group compared to the control group.

It has been reported that increased expression of *CAMP* in human breast cancer (19). Additionally, *NMB* exhibited increased expression in various human tumors, including breast (20, 21), lung (22, 23), colon (24), and ovary (25). In the present study, a decreased expression of both genes was identified, and there are no confirmed reports related to expression in lymphoma or veterinary science. This discrepancy may be attributed to species- or tumor-specific differences, and further research is needed.

**Figure 2:**
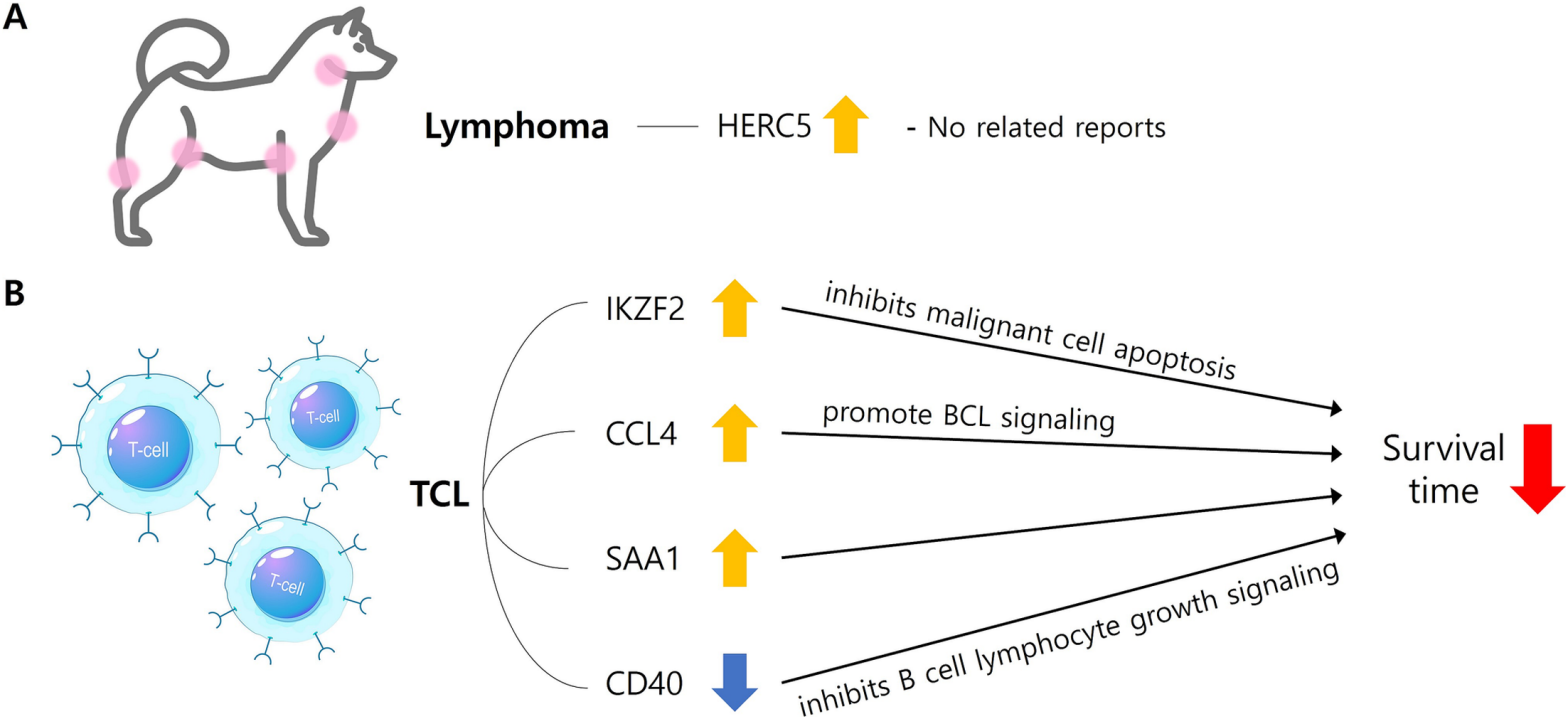
The mechanism by which significant DEGs identified in the lymphoma **(A)** and TCL group **(B)** are involved in characteristics. In lymphoma group, *HERC5* gene was upregulated (yellow arrow), but there were no related reports with lymphoma in dogs **(A)**. In TCL group, *IKZF2*, *CCL4*, and *SAA* were upregulated (yellow arrow) and *CD40* was downregulated (blue arrow), and each was reported to be involved in a shorter survival time through the above mechanisms **(B)**. DEGs, Differentially expressed genes; TCL, T-cell lymphoma; BCL, B-cell lymphoma; HERC5, HECT and RLD domain containing E3 ubiquitin protein ligase 5; IKZF2, IKAROS family zinc finger 2; CCL4, C-C motif chemokine ligand 4; SAA1, Serum amyloid A1; CD40, Cluster of differentiation 40 molecule.

Regarding the enriched pathways in the lymphoma group, it is noteworthy that the T-cell receptor signaling pathway (26, 27) and the PI3K-Akt signaling pathway (28, 29) have been associated with lymphoma in previous literature. Among the DEGs, *IKZF2* identified as one of the most prominently upregulated genes. This gene has been reported to play a critical role in T-cell development, differentiation, and function as a transcription inhibitor (30). A recent study highlighted the upregulation of IKZF2 in human cutaneous TCL and proposed its potential as a target gene for treatment (Figure 2B) (30), as well as in T-cell acute lymphoblastic leukemia in humans (31). Furthermore, in this study identified that *IKZF2* was also upregulated in the canine TCL group when compared to the canine BCL group. This finding suggests that *IKZF2* may serve as a relevant gene associated with poorer clinical outcomes in TCL rather than BCL in dogs.

There are reports indicating high concentrations of CCL4 are associated with reduced overall survival and progression-free survival (32, 33). High expression of SAA is significantly related to extranodal lesions, elevated LDH (Lactate Dehydrogenase) levels, and high NCCN-IPI (National Comprehensive Cancer Network-International Prognostic Index) scores, with shorter survival times compared to the control group (34) in human diffuse large B-cell lymphoma (DLBCL), indicating a poor prognosis (Figure 2B). Furthermore, it has been reported that *IL1R2* is upregulated in breast cancer, and higher expression is correlated with lower survival rates in humans (35). These findings suggest that these three genes may contribute to the characteristics of canine TCL.

Among the downregulated DEGs, *PLEKHA5*, *GRK4*, *MYRIP*, and *CD40* have established associations with various human cancers (36–39). *PLEKHA5* (39) has been related with melanoma, while *GRK4* (36) and *MYRIP* (38) have been associated with hepatocellular carcinoma in humans. These genes exhibit to have low expression levels in human cancers, and their downregulation has been correlated with the promotion of tumor metastasis and poorer clinical outcomes. Furthermore, *CD40* is downregulated in human DLBCL, and lower expression linked to decreased survival rates (37). This suggests that CD40 may be a gene of significance concerning poorer clinical outcomes in TCL as opposed to BCL in dogs (Figure 2B). Among the enriched pathways identified in the canine TCL group compared to the canine BCL group, human T-cell leukemia virus 1 infection (40–42) and transcriptional misregulation in cancer (43) have previously been associated with lymphoma. The roles of the confirmed DEGs identified in this study are summarized in Table 7 (44–53).

**Table 7:** The role of DEGs.

Gene	Role	Ref
*MX1*	GTP release and cellular antiviral protein metabolism	(44)
*HERC5*	E3 ubiquitin ligase function, interferon signaling mediation, ISGylation	(45)
*OAS1*	Apoptosis induction, IFN-α signal response enhancement, gene regulation, immune receptor regulation, autophagy	(46, 47)
*DDX58*	Type I interferon production, antiviral response, innate immune response	(16)
*CAMP*	Tumor growth promotion, invasion, angiogenesis initiation, immune cell recruitment, wound healing promotion	(48–50)
*NMB*	Breast cancer cell growth control	(51)
*IL7R*	Tumor growth inhibition, immune cell proportion regulation in tumor microenvironment	(18)
*IKZF2*	T cell development, differentiation, transcription inhibition	(30)
*CCL4*	Inflammatory CC chemokine subfamily, inflammation response	(49)
*IL1R2*	Cytokine decoy receptor encoding, interleukin-1 receptor family	(35)
*SAA1*	SAA1 protein encoding, acute-phase protein production by hepatocytes in response to infection, injury, malignancy	(52)
*PLEKHA5*	Brain development involvement, unknown function in human cancer	(39)
*GRK4*	G protein-coupled receptor kinase subfamily encoding, Ser/Thr protein kinase family, hypertension association	(36)
*MYRIP*	Cytoskeletal protein binding activity, protein kinase A binding, small GTPase binding	(38)
*CD40*	TNF-receptor superfamily protein receptor encoding	(53)

Ref, Reference; GPT, Guanosine triphosphate; IFN-α, Interferon- alpha; TNF, Tumor necrosis factor; G-protein, Guanine nucleotide-binding protein.

This study faced several limitations including small sample sizes and discrepancies in group numbers, hindering comprehensive variance analysis of the entire cohort. The exclusive focus on one species (dog) and a single breed (Beagle), within the control group restricted genetic diversity, contrasting with the actual clinical data involving client-owned dogs. Furthermore, the lack of longitudinal follow-up data for the study subjects prevented establishing prognostic evaluations based on differences in gene expression. Despite these limitations, this pioneering study is the first to conduct transcriptome profiling of both normal and lymphoma tissues in dogs, including a comparative analysis between TCL and BCL.

In conclusion, the current study demonstrates that the regulation of specific genes, which has not previously been reported in canine TCL, may function as a prognostic indicator. A comprehensive understanding of these regulatory mechanisms may facilitate the quality of care provided to dogs affected by lymphoma, thereby potentially extending the average lifespan of companion animals. Consequently, this study is considerable significance for both pet owners and their canine companions.

## Data Availability

The datasets presented in this study can be found in online repositories. The names of the repository/repositories and accession number(s) can be found below: https://www.ncbi.nlm.nih.gov/geo/, GSE285369.
