# Supplementary material for: Sequential transcriptome profiling: comparative analysis of normal and canine lymphoma preceding detailed T-cell and B-cell subtype comparison
Source: Front Vet Sci. 2025 Jan 22;11:1473421. doi: 10.3389/fvets.2024.1473421 (PMC11795822; doi:10.3389/fvets.2024.1473421)
Supplement: Supplementary file 1 [file Data_Sheet_1.docx]

**Supplementary Material**

1. **Supplementary Figures and Tables**
   1. **supplementary Figures**


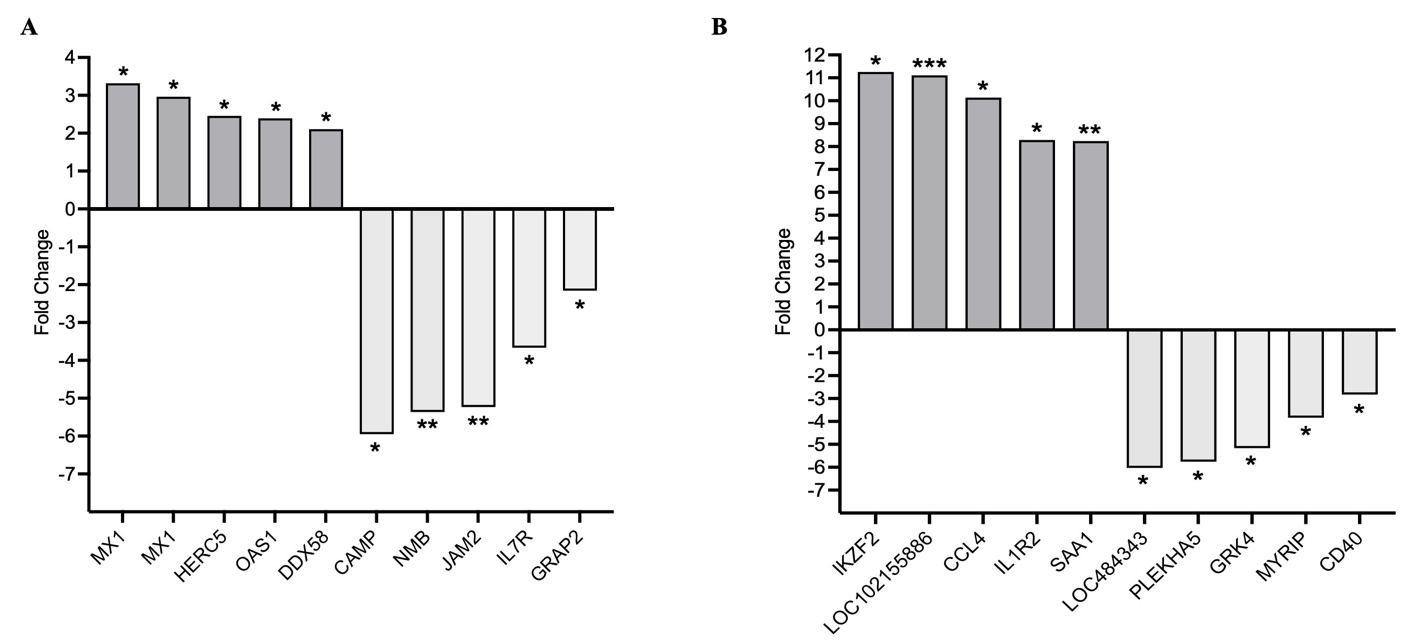


**Supplementary Figure 1.** Image of the top five upregulated DEGs and downregulated DEGs in the lymphoma group compared with the control group (A) and in the TCL group compared with the BCL group (B). **P* < 0.05, ***P* < 0.01, and ****P* < 0.001.

- 1. **Supplementary Tables**

**Supplementary Table 1.** Staging system for canine lymphoma according to the World Health Organization's TNM classification.

| **Stage** | **Explanation** |
| --- | --- |
| I | One lymph node involved or lymphoid tissue in a single organ  (Excluding bone marrow) |
| II | Involvement of many lymph nodes in a regional area |
| III | Generalized lymph node involvement |
| IV | Liver and spleen involvement ± stage III |
| V | Bone marrow involvement or extranodal disease |
| **Substage** | **Explanation** |
| a | Absence of systemic signs |
| b | Presence of systemic signs |
